# Free Radical-Mediated Grafting of Natural Polysaccharides Such as Chitosan, Starch, Inulin, and Pectin with Some Polyphenols: Synthesis, Structural Characterization, Bioactivities, and Applications—A Review

**DOI:** 10.3390/foods12193688

**Published:** 2023-10-08

**Authors:** Wenting Zhang, Jian Sun, Qiang Li, Chanmin Liu, Fuxiang Niu, Ruixue Yue, Yi Zhang, Hong Zhu, Chen Ma, Shaoying Deng

**Affiliations:** 1 Xuzhou Institute of Agricultural Sciences, Jiangsu Xuhuai District, Xuzhou 221131, China; 20171002@jaas.ac.cn (W.Z.); niufuxiang@sina.com (F.N.); yueruixue_1983@163.com (R.Y.); zhangyijsnu@163.com (Y.Z.); zhuh-135@163.com (H.Z.); 15138891510@163.com (C.M.); ddshaoying18@163.com (S.D.); 2 School of Life Sciences, Jiangsu Normal University, Xuzhou 221116, China; cm9009@126.com

**Keywords:** polyphenol-grafted polysaccharides, free radical-induced grafting method, chemical preparation, function

## Abstract

Polyphenols and polysaccharides are very important natural products with special physicochemical properties and extensive biological activities. Recently, polyphenol-polysaccharide conjugates have been synthesized to overcome the limitations of polysaccharides and broaden their application range. Grafted copolymers are produced through chemical coupling, enzyme-mediated, and free radical-mediated methods, among which the free radical-induced grafting reaction is the most cost-effective, ecofriendly, safe, and plausible approach. Here, we review the grafting reactions of polysaccharides mediated by free radicals with various bioactive polyphenols, such as gallic acid (GA), ferulic acid (FA), and catechins. A detailed introduction of the methods and their mechanisms for free radical-mediated grafting is given. Structural characterization methods of the graft products, including thin-layer chromatography (TLC), ultraviolet-visible (UV-vis) spectroscopy, Fourier transform infrared (FT-IR) spectroscopy, nuclear magnetic resonance (NMR) analysis, and X-ray diffraction (XRD) are introduced. Furthermore, the biological properties of polyphenol-polysaccharide conjugates are also presented, including antioxidant, antibacterial, antidiabetic, and neuroprotection activities, etc. Moreover, the potential applications of polyphenol-polysaccharide conjugates are described. Finally, the challenges and research prospects of graft products are summarized.

## 1. Introduction

**Figure 1:**
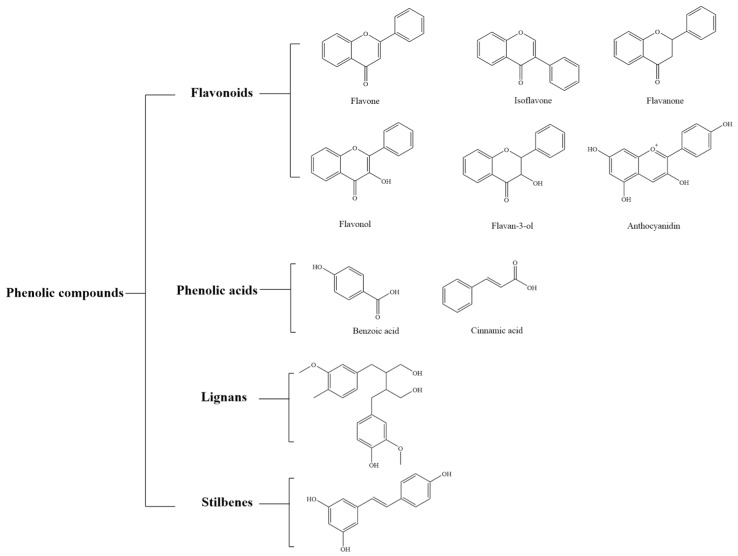
Types of natural polyphenols.

Polyphenols are a series of natural compounds based on aromatic rings with multiple hydroxyl groups. They account for 40% of organic substances. They mainly exist in plants, such as tea, grass, pomegranates, apples, grape seeds, oranges, cocoa beans, purple sweet potatoes, and peppers [1]. The four main structural types of natural polyphenols include flavonoids, phenolic acids, lignans, and stilbenes [2]. Among them, flavonoids and phenolic acids account for 60% and 30%, respectively (Figure 1).

Polyphenols have antioxidant, anti-inflammatory, anticancer, and antibacterial activities and the ability to prevent degenerative diseases [3,4,5,6]. They have extensive applications in the fields of household chemical products, medicine, food additives, agriculture, and functional polymer materials.

Polysaccharides are natural macromolecular compounds composed of at least 20 monosaccharide units [7,8,9]. Natural polysaccharides are mainly divided into microbial polysaccharides (fungal and bacterial polysaccharides), animal polysaccharides, and plant polysaccharides. Plant polysaccharides include lower plant polysaccharides (mainly algae) and higher plant polysaccharides. Polysaccharides, along with proteins and polynucleotides, are very important biological macromolecules in organisms, each of which plays a different role in maintaining life activities.

Polysaccharides are the main active substance of many natural products. Foodborne polysaccharides have functional activities such as antioxidant [10,11,12], antitumor [13,14], hypoglycemic [15,16], immune regulation [17,18], and cholesterol-lowering activities [19,20]. Compared with chemically synthetic drugs, they have lower toxicity and side effects [21]. Therefore, the research on polysaccharides is now receiving increasing attention. Among them, the natural polysaccharides that have been extensively studied are chitosan and its structural modifications. Researchers combine chitosan with other biopolymers to maximize its benefits and reduce its limitations, such as poor mechanical and thermal stability [22,23,24].

Studies have shown that polyphenols can be combined with polysaccharides, and the formed complexes not only have the merits of both but also generate synergistic effects, such as enhanced physical, chemical, and functional properties. Therefore, they show great potential for applications in cosmetics, food, and medicine [25]. For instance, research has indicated that consuming foods rich in both polyphenols and polysaccharides was more beneficial for body functions, such as reducing plasma and liver lipid levels [26,27]. Therefore, their synergism potential may enhance the efficacy of polysaccharides and polyphenols as healthy ingredients in foods.

Non-covalent and covalent interactions are the two main modes of interaction between polyphenols and polysaccharides. Using noncovalent interactions, polyphenols can approach the surface of polysaccharide molecules through hydrophobic bonds, and polyphenol molecules enter hydrophobic bags to undergo multi-point hydrogen bonding [28]. The degree of polymerization of polyphenols, the number of hydrophobic groups, the chain conformation, and the hydrophobicity of polysaccharides affect the binding [29]. For example, the structural characteristics of anthocyanins, such as the degree of polymerization and molecular size, as well as the structural and conformational characteristics of pectin, affect their binding with pectin. The affinity between citrus pectinase and high polymerization degree procyanidins was stronger than that of low polymerization degree procyanidins. The interaction between methylated homogalacturonans and a high degree of polymerization of procyanidins was found to be mainly through hydrophobic interactions [30]. Additionally, the association between proanthocyanidin molecules with a long chain length and highly methylated pectin produced a stronger correlation. In addition, temperature, pH, reaction time, and reactant concentration can also impact interactions [31]. For example, the interaction between pectin and two anthocyanins with different hydroxyl groups was studied through molecular dynamics simulations. The results indicated that both types of pectin had stronger interactions with anthocyanins via three hydroxyl groups [32]. Another study showed that lower pH and substrate concentrations both resulted in stronger adsorption of blueberry pectin and anthocyanins. The speculated reason was that the decrease in pH led to a weakened interaction between them, while the hydrophobic interaction increased, and the hydrogen bond tended to be constant, resulting in a higher binding degree between anthocyanins and pectin. In a word, small changes in pH, concentration, and other external factors may largely influence the polyphenol-polysaccharide binding and functional characteristics [33].

Covalent bonds are a type of chemical bond that has stronger forces than non-covalent bonds. Thus, polyphenols and polysaccharides possess more stable structures through covalent bonds. In medicinal plants and crops, covalently linked polyphenol-polysaccharide conjugates were also abundant [34,35,36,37]. Nevertheless, the difficulty in the separation and the complexity of structural features (especially three–dimensional structure) make the specific characterization of the complex very difficult. Therefore, people usually prefer to use chemical methods to graft multiple polyphenols onto polysaccharides artificially [38,39,40,41]. 

Methods of introducing different types of polyphenols onto the polysaccharide structure include chemical coupling, enzyme-mediated method, and free radical grafting method. 

The chemical coupling of polysaccharides and polyphenols is often achieved using the bifunctional coupling agent carbodiimide [42,43,44]. Among them, EDC is the most widely used coupling agent. For example, caffeic acid–chitosan conjugate [45] and chlorogenic acid–chitosan conjugate [46] were synthesized using the EDC-mediated method. However, the process of this reaction is accompanied by a large number of toxic and harmful EDC reagents [47]. Therefore, this method is not conducive to human health and cannot be widely used in food and medicine.

Laccase is the most widely used in the grafting reaction of polyphenols and polysaccharides in recent years [48]. Natural polyphenols are grafted onto polysaccharide molecules through enzyme catalysis in a grafting environment that is relatively mild. The process does not need to be carried out in organic solvents. Compared with the chemical coupling method, enzyme-mediated biocatalytic synthesis is superior in terms of synthesis efficiency, specificity of reaction conditions, and environmental protection [48,49]. However, in the catalytic process, polyphenol oxidase oxidizes the effective functional group of polyphenols, which inevitably affects and inhibits the excellent performance of polyphenols [46]. Therefore, identifying how to avoid this inhibition will be an important breakthrough in enhancing the biochemical activity of the conjugate. 

Recently, the free radical grafting approach has received extensive attention in the covalent modification of polysaccharides by polyphenols [50,51,52,53]. Among the three methods of polyphenol grafting modification of polysaccharides, the free radical grafting method is considered the ideal method, which is simple, safe, and environmentally friendly. Using this method to prepare polysaccharide polyphenol grafting products has the most practical value. In Section 2, the free radical grafting method was introduced in detail.

This paper summarizes the synthesis, structural analysis, bioactivities, and potential applications of polyphenol-polysaccharide graft copolymer mediated by free radicals in order to make contributions to the more accurate design and targeted use of polyphenol-polysaccharide conjugates in the future. 

## 2. Free Radical-Mediated Grafting Method

Free radical initiator systems include ascorbic acid (Vc)–hydrogen peroxide (H_2_O_2_) redox pairs and some peroxide compounds. Among them, the Vc/H_2_O_2_ redox system has been extensively used in the grafting reaction of various polyphenols and polysaccharides since the first successful application of Vc and hydrogen peroxide redox, which was the synthesis of catechin-grafted chitosan by Curcio et al. [54] in 2009. The method of using Vc/H_2_O_2_ redox pair to initiate the grafting reaction has several advantages: first, the reaction reagents for generating free radicals are lower cost than either carbodiimide or enzymes; second, Vc/H_2_O_2_ redox is less toxic than carbodiimide. Additionally, this chemical reaction does not require heating, which effectively avoids the destruction of the phenolic structure, leading to higher yields and lower toxicity of the products than with other methods [55].

**Figure 2:**
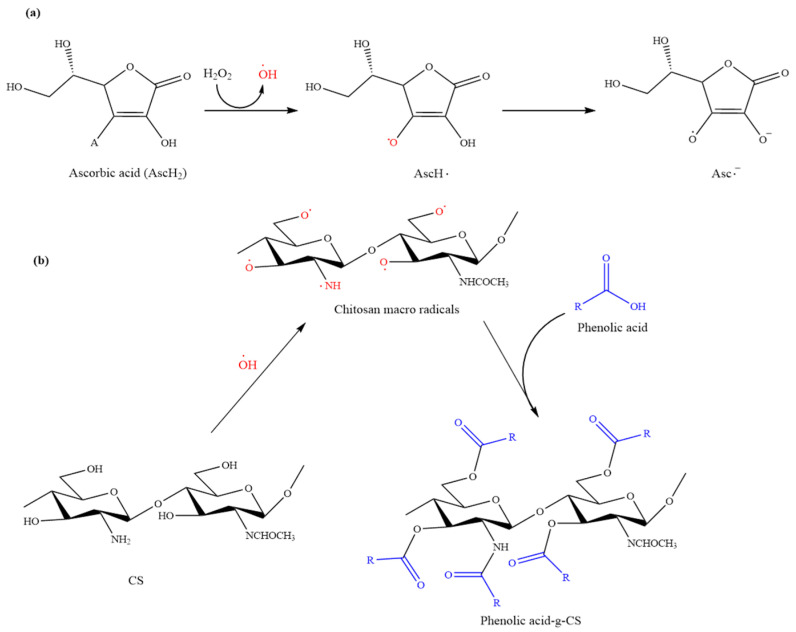
Possible mechanism for the synthesis of polyphenol-g-CS by Vc/H_2_O_2_ redox pair-mediated grafting method. (**a**) Procedure for the generation of •OH via Vc/H_2_O_2_ redox pair. (**b**) Conjugation of polyphenol onto CS by • OH-mediated grafting reaction [56].

In addition, this method can also be used for polysaccharides without amino groups. Therefore, it greatly increases the diversity of polyphenol-polysaccharide grafts. Consequently, the Vc/H_2_O_2_ redox pair-mediated grafting reaction is not only greener and economical than the chemical coupling method. It also avoids the oxidation of polyphenols in enzyme-mediated reactions. Although the exact mechanism of this reaction still needs to be confirmed through more experiments, most researchers believe that its possible mechanism is as shown in Figure 2, in which polysaccharides are represented by chitosan (CS).

In this reaction, Vc exists in the form of diacid (A_SC_H_2_) in the solution, which reacts with H_2_O_2_ to generate ·OH and the spin-stabilized tricarbonyl ascorbic acid radical (AscH·). Owing to AscH· being acidic, it is not easy to be protonated and usually exists in the form of semi-dehydrogenated ascorbic acid radical (AscH^−^). Subsequently, the generated molecules can snatch hydrogen atoms from the polysaccharide molecules, forming polysaccharide macromolecular free radicals. Finally, the polyphenol monomer near the reaction site becomes the receptor of the polysaccharide macromolecule free radical, thus forming the polysaccharide–polyphenol graft copolymer. Therefore, many researchers believe that ·OH is the key to initiating the graft copolymerization between polysaccharides and polyphenols. However, there is still no direct experimental evidence for this statement. 

To clarify the exact mechanism of this grafting reaction, Liu et al. [57] compared the free radicals generated in Vc/H_2_O_2_ oxidation–reduction system with the ·OH generated in an Fe^2+^/H_2_O_2_ reduction system. The Fe^2+^/H_2_O_2_ oxidation–reduction system is a classical Fenton reaction, which produces hydroxyl radicals (Fe^2+^ + H_2_O_2_ → Fe^3+^ + OH^−^ +·OH). The free radicals (·OH) generated by these two redox systems can be compared. First, in an experimental design, free radicals were generated in the Fe^2+^/H_2_O_2_ and Vc/H_2_O_2_ redox systems and verified by an electron paramagnetic resonance technique. Then, caffeic acid (CA) was grafted onto CS through Fe^2+^/H_2_O_2_ and Vc/H_2_O_2_ redox systems, respectively. Lastly, structural analysis of the different grafted products was conducted using various characterization methods. Results indicated that only Asc^−^ was detected in the Vc/H_2_O_2_ system. The reaction between Asc^−^ and CS produced new substances (novel carbon-centered radicals), whereas no radicals were detected when ·OH reacted with CS. The study suggested the reaction between CS and CA in the Vc/H_2_O_2_ redox system was mediated by Asc^−^ rather than ·OH. Asc^−^ generated in Vc/H_2_O_2_ redox system abstracted hydrogen atom from CS and produced carbon-centered radicals along CS chains, resulting in a reduction of intermolecular hydrogen bonds.

Therefore, the free radical–initiated graft reaction is a new development and environmentally protective approach to synthesizing polyphenol-polysaccharide conjugates. In this reaction process, an organic solvent is not involved, which avoids the oxidation damage of polyphenolic structure. It is a “green” preparation method and maintains the excellent chemical properties of polyphenols. 

After the grafting reaction is completed, the general purification steps are as follows [51,53,58]. Firstly, the resulting mixture is centrifuged for 20–30 min to remove unreacted free polyphenols and precipitate overnight with 4 volumes of 95% ethanol at 4 °C at once. Then, the precipitate is collected through filtration or centrifugation steps, dissolved again in distilled water, and dialyzed with distilled water (MWCO: 6–8 kDa) for 48 to 72 h. Finally, the dialysate is lyophilized to obtain a relatively pure graft conjugate solid. In addition, if the sample still contained unreacted polysaccharides, a column separation process could be performed to remove a small amount of excess polysaccharides.

## 3. Structural Characterization

### 3.1. Thin-Layer Chromatography 

Thin-layer chromatography (TLC) is often used for the separation or purification of mixtures of chemical compounds due to its advantages of being convenient, economical, and easy to use, and it does not require large volumes of organic solvent [59]. To verify the presence of free polyphenols in polyphenol-polysaccharide conjugates, TLC analysis is usually performed. 

Bai et al. [60] separated gallic acid (GA), *O*-carboxymethyl chitosan (*O*-CMCS), and synthesized GA grafted *O*-CMCS (GA-g-CMCS) onto a silica gel GF254 plate by using trichloromethane–ethyl acetate–acetic acid (50:50:1, *v*/*v*) as expansion agent. First, GA, *O*-CMCS, and GA-g-CMCS were individually developed on a silica gel plate. Then, the developed plate was fumigated with iodine vapor for 10 min. Finally, the results showed that no spots of GA were observed on the developed TLC plate, indicating that GA had participated in the reaction and grafted onto *O*-CMCS. Similar experimental plans were established by Chatterjee [61], Liu [57], and Cho et al. [62] applying chloroform–ethyl acetate–acetic acid (50:50:1) or butyl alcohol–deionized water–acetic acid (50:40:1) to the developing solvent, respectively. To enhance the color rendering effect and hold the color rendering for a period of time, the plate is often exposed to 30% H_2_SO_4_ or iodine vapors, and spots with different migration lengths emerge on it. Generally, phenolic compounds have a higher R_f_ (distance of analyte migration/distance of mobile phase migration) value than grafted products. Therefore, phenolic compounds migrate some distance on the TLC plate, while polyphenol-polysaccharide conjugates stay at the baseline. More TLC technologies, such as high-performance thin-layer chromatography (HPTLC), are likely to be used in the separation and preparation of different polyphenol-polysaccharide conjugates in the future [63].

### 3.2. Ultraviolet-Visible Spectroscopy

Ultraviolet-visible (UV-vis), an electron transition spectrum with an absorption wavelength range of 200–400 nm, can be applied to identify conjugation and certain characteristic functional groups, substance content, and isomers. Ordinarily, polyphenolic compounds exhibit one or two significant absorption bands, as there is generally not one aromatic ring in their structure. On the contrary, except for a few natural polysaccharides such as inulin in which a clear absorption peak at 210 nm appears in the spectrum [64], most polysaccharides do not exhibit absorption bands in the wavelength range of 200–400 nm due to the lack of chromophore. Therefore, the successful graft reaction can be determined by the analysis and comparison of the UV spectra of polyphenols, polysaccharides, and their grafting products. 

For instance, FA has two signals at about 294 nm and 321 nm, which have been indicated to the conjugated system of the FA benzene ring. After conjugation with carboxylic curdlan, the grafted products showed two characteristic absorption peaks near 293 and 319 nm, indicating that FA was grafted onto the carboxylic acid curdlan successfully [65]. Notably, most researchers have found that the UV-vis absorption peaks of polyphenol-grafted polysaccharides shifted toward longer wavelengths. This phenomenon is attributed to the relative configuration between the energy levels of the conjugated system, which is relatively compact, and the energy required for the n→π* and π→π* electronic transitions [58,60,66,67]. However, Zeng et al. [64] found that the characteristic peaks at 209 and 272 nm in the spectrum of the banana condensed tannin–inulin conjugate (BCT-g-inulin) shifted to shorter wavelengths (207 and 271 nm). This change may have been caused by active groups of BCT, which decreased the π→π* electronic transition energy gap and probability of electronic transition [68]. Additionally, the UV-vis absorption intensity of polyphenol-polysaccharide conjugates was positively correlated with the grafting rate [69,70].

### 3.3. Fourier Transform Infrared Spectroscopy

Fourier transform infrared (FT-IR) spectroscopy is a characterization method for inferring the functional groups of organic substances. It is mainly applied to analyze the composition of unknown organic substances qualitatively and can obtain important information, such as the composition of the main covalent bond and functional groups. By comparing the differences in the FT-IR spectra of polysaccharides and grafted products, the structural characteristics of the polyphenol-polysaccharide conjugates can be inferred [51,52,53].

Usually, several new bands are produced in covalently linked conjugates. For example, new characteristic absorption bands appeared at about 1514 cm^−1^ in the IR spectra of ferulic acid-grafted curdlan conjugates (Cur-g-FA) in comparison with those of curdlan. This illustrated that covalent linkages occurred between FA and the hydroxyl groups of curdlan, thus further implying that FA was grafted onto the main chain successfully. Additionally, the grafting effect of the coupling compound can be calculated by tracking the changes in peak strength. For example, the peak intensity of Cur-g-D-FA at 1514 cm^−1^ was slightly higher than that at 1516 cm^−1^ for Cur-g-FA. It showed that the grafting effect of Cur-g-D-FA was better than that of Cur-g-FA [50].

### 3.4. Nuclear Magnetic Resonance Analysis

Nuclear magnetic resonance (NMR) spectroscopy is the main analytical technology for obtaining structural information of organic compounds and biological macromolecules. It can provide valuable structural information about the entire molecule through a series of analytical tests. As summarized in Table 1, the molecular structure of polyphenol-grafted polysaccharides has been characterized by ^1^H NMR and ^13^C NMR [50,66,70].

Generally, the structure of polyphenol-polysaccharide conjugates retains most of the structural information of both the polyphenols and the polysaccharides. Therefore, the chemical shift and peak splitting of the grafted product in the ^1^H NMR spectrum generally reflect the chemical shift characteristics of the polyphenol moiety and polysaccharide main chain. For example, the ^1^H NMR spectrum of catechin grafted *Tremella fuciformis* polysaccharide (catechin-g-TPS) displayed the whole characteristic proton signals of TPS (*δ* = 5.18 ppm, *δ* = 5.10 ppm, *δ* = 4.35 ppm, *δ* = 2.02 ppm, *δ* = 1.16 ppm). In addition, new hydrogen signals were observed in the ^1^H NMR spectrum of the graft (*δ* = 6.80 ppm, *δ* = 6.60 ppm), which proved the success of the grafting reaction [52].

^13^C NMR is also a characterization method for the structure of polyphenol-polysaccharide conjugates. The ^13^C NMR spectrum of CS showed signals at 57.8 ppm (C-2), 61.1 ppm (C-6), 75.5 ppm (C-3), 83.2 ppm (C-5), 105.3 ppm (C-4), 174.2 ppm (C-7), and 23.7 ppm (C-8). The carbonyl and methyl groups of N-acetylglucosamine showed signals at 174.2 ppm (C-7) and 23.7 ppm (C-8), respectively. Compared with CS, phenolic acid–CS conjugates showed new peaks near 150 ppm. Based on the peaks of carbon atoms, the peaks were assigned to the C=C double bond of polyphenol. Furthermore, for the formation of a carbonyl group (C=O) between the carboxyl group of phenolic acid and CS, a signal enhancement of 174.2 ppm (C-7) occurred. These results once again confirmed the covalent binding between phenolic acids and CS chains [66].

In addition, two-dimensional (2D) NMR technologies are suitable for the structural identification of complex natural products and biological macromolecules [71,72,73,74,75,76]. It can provide the connection relationship and spatial configuration between H-H, C-H, and C-C, which is conducive to the spectral analysis of complex compounds. Two dimensional NMR spectroscopy has also been widely used for polysaccharide, polyphenol, and polyphenol-polysaccharide conjugate analyses.

### 3.5. Crystallinity Analysis

X-ray diffraction (XRD) is a research method for obtaining information about the composition, internal atomic structure, or morphology of materials through analysis of their X-ray diffraction patterns [77]. It can serve as a supplementary method for analyzing the crystallinity of polyphenol-polysaccharide conjugates. Usually, it is possible to determine whether a material is a crystalline material on the basis of the shape of the XRD peak. Sharp, narrow peaks indicate that the material is in a crystalline state, while wide peaks reflect an amorphous state [38,52].

Liu et al. [53] determined the crystallographic structures of CS, N, O-carboxymethyl CS (NOCC), and NOCC grafted copolymers by XRD. The results indicated that the diffraction pattern of NOCC at 2*θ* = 20° displayed a wider characteristic peak than that of the original CS, indicating that NOCC and CS were amorphous and semi-crystalline, respectively. However, the grafts of GA, CA, and FA with NOCC exhibited broader and weaker peaks at 2*θ* = 23.1°, 23.6° and 22.4°, separately, confirming a successful graft. These results indicated that the inter- and intramolecular hydrogen bonds of the original NOCC had significantly decreased after grafting, resulting in the increasing looseness of the packing structure and improving water solubility of grafted products. Consistent results have also been reported by other authors [66,67,78].

### 3.6. Scanning Electron Microscope

Scanning electron microscopy (SEM) can characterize the microscopic morphology of substances and field-emission scanning electron microscope (FE-SEM) can obtain highly three-dimensional and faithful information on the surface microstructure of the sample. Currently, SEM and FE-SEM have been used to observe the surface morphology of polyphenol-polysaccharides [51,52,60,79,80,81].

As reported, apparent differences in the surface morphology of polysaccharides were discovered once grafted with polyphenols. Polysaccharides usually showed a smooth surface, while grafted polymers had a much rougher surface, which was attributed to the hydrogen bonds [52,64,65,79]. Wang et al. [65] observed that the appearance of carboxylic curdlan copolymer changed after FA grafting and exhibited a relatively rough and fractured surface structure. This was because introducing phenolic substances could disrupt the integrated structure of polymers [82]. However, there has also been the opposite situation. Wang et al. [51] demonstrated that native pectin had a rough and compact flake structure comparatively. Yet, pectin-g-FA presented a flake-like network with a comparatively smooth and wide surface. The results indicated that there was a significant correlation between the surface characteristics of polyphenol-polysaccharide conjugates and the characteristics of natural polysaccharides [83].

## 4. Gallic Acid–Polysaccharide Conjugate

### 4.1. Gallic Acid

Gallic acid (GA), chemically known as 3,4,5-trihydroxybenzoic acid, is a natural polyphenolic acid with a simple chemical structure. It is a secondary metabolite of plants and is found in various vegetables, fruits, and other plants [84,85]. GA has multiple bioactivities, including antioxidant, antitumor, antibacterial, and liver-protective functions [86,87,88,89]. It has preventive and therapeutic effects on the cardiovascular and nervous systems and against diabetes, liver fibrosis, tumors, and other diseases, thus providing broad application prospects for disease treatment [90]. The gallic acid–polysaccharide (GA-g-PS) conjugate is one of the most studied conjugates, and the biological activities of GA-g-PS have been widely reported and have shown many potential applications.

### 4.2. Biological Activity

#### 4.2.1. Antioxidant Activity

The metabolic reactions of the human body produce various free radicals and reactive oxygen species (ROS). Their excessive accumulation can result in oxidative damage to cells and lead to aging, atherosclerosis, cancer, and other diseases [91]. In addition, oxidation causes quality degradation of fruits, vegetables, and fruit juices during storage and transportation, affecting the visual and taste experience [92]. Therefore, the study of antioxidant substances has attracted wide attention. Considerable evidence has demonstrated that grafting GA onto polysaccharides significantly enhanced the antioxidant activity of polysaccharides [52,66,67,93].

The antioxidant activities of GA grafted CS (GA-g-CS) or N,O-carboxymethyl CS (GA-g-NOCC) conjugates have been studied through different free radical scavenging assays [53,64,69]. Results showed that the grafting ratios of GA-g-NOCC and GA-g-CS were determined as 45.8 mg CAE/g and 72.27 mg CAE/g, respectively. The antioxidant activity decreased in the order of GA-g-NOCC > NOCC > CS [53]. At 0.4 mg/mL, the 2,2-diphenyl-1-picrylhydrazyl (DPPH) and 2,2′-azinobis-(3-ethylbenzothiazoline-6-sulfonic acid) (ABTS) radical scavenging activities of GA-g-CS was the same as that of free GA. In addition, the graft product showed superior cytocompatibility against RAW264.7 mouse macrophages. And GA-g-CS also showed the formation of intracellular ROS in a time- and dose-dependent manner in RAW264.7 mouse macrophages [62]. Zhang et al. [94] functionalized CS with GA by free radical initiation and other means. The obtained GA-g-CS were further developed into films by casting and showed DPPH radical scavenging ability. The antioxidant level of the film was positively correlated with the grafting rate [95]. This implied that this material could further develop as an active antioxidant packaging film.

#### 4.2.2. Antibacterial Activity

With the increasing resistance to antibiotics, research and development of new antibacterial agents is urgent. Particularly in the food industry, there is a demand for non-toxic and safe natural antibacterial agents.

Some GA-g-PS conjugates have been shown to have significant antibacterial properties. According to Singh et al. [96], the antibacterial activity of GA grafted chitin–glucan (GA-g-glucan) complexes, which was calculated that 50% of GA was grafted with chitin-glucan complex, were studied against Gram-positive and Gram-negative bacteria, *Bacillus subtilis*, and *Escherichia coli*, respectively. Due to the diversity of Gram-positive and Gram-negative bacteria, the GA-g-glucan complex only had an obvious inhibitory effect on the growth of *Bacillus subtilis* and *Escherichia coli*. However, the mechanism of the antibacterial effect of the graft copolymer still needs further exploration.

## 5. Ferulic Acid–Polysaccharide Conjugate

### 5.1. Ferulic Acid

Ferulic acid (FA) is a derivative of cinnamic acid, which is commonly found in plant tissues [97,98,99]. They are easily absorbed and metabolized by the human body and have low toxicity and safety. Their medicinal value is receiving increasing attention.

FA compounds have a wide range of biological activities, including renal protective, antibacterial, antidepressant, anti-apoptotic, antiviral, and anticancer activities [100,101,102,103]. In addition, FA is a reduction agent and has a strong scavenging effect on free radicals, such as peroxides and superoxide compounds [104,105]. Grafting FA onto polysaccharides, such as CS, carboxymethyl CS, and pectin can enhance the biological function of polysaccharides and the physicochemical properties of FA [51,53,58,106,107]. For the last few years, the coupling of FA and polysaccharides has attracted increasingly more attention.

### 5.2. Biological Activity

#### Antioxidant Activity

As reported by Cai et al. [50], water-soluble curdlan products (Cur and Cur-D) were prepared, and FA-grafted Cur conjugates (Cur-g-FA and Cur-D-g-FA) were prepared by Vc/H_2_O_2_ redox systems under an inert atmosphere. The grafting ratios of Cur-g-FA and Cur-D-g-FA were tested to be 99.30 mg FA/g and 102.93 mg FA/g, respectively. FA-g-Cur conjugates indicated significantly enhanced scavenging activity for DPPH radicals (*p* < 0.05) compared with native Cur-D. Their effective DPPH radical scavenging ability was mainly dependent on the grafted FA portion of the Cur backbone. Furthermore, the DPPH radical scavenging activity of the grafted products was proportional to the grafting rate. In addition, Cur-*g*-FA and Cur-D-*g*-FA had significantly better Trolox equivalent antioxidant capacities (TEACs) compared to native Cur-D, indicating that the combination of them enhanced the antioxidant activity.

The same group [51] also grafted FA onto pectin (UP30 and UP60) through a free radical-mediated grafting process, and pectin-g-FA, UP30-g-FA, and UP60-g-FA conjugates were prepared with the grafting ratios of 65.43 mg FA/g, 82.55 mg FA/g and 75.82 ± 0.89 mg FA/g, respectively. The conjugates possessed prominent DPPH radical scavenging ability (IC_50_: 0.32–0.89 mg mL^–1^) and antioxidant capacity (TEAC: 100.02–153.42 μmol Trolox/gsample; FRAP: 166.41–270.27 μmol FeSO_4_ g/sample). The research results indicated that FA-grafted pectin could serve as an excellent antioxidant agent and showed great potential in many fields, such as medicine.

Liu, et al [53,66] grafted FA onto N, O-carboxymethyl CS (NOCC), and CS, respectively, and obtained grafted products FA-g-NOCC and FA-g-CS. The grafting ratios of FA-g-NOCC and FA-g-CS were 36.7 mg FA/g and 66.7 mg FA/g, respectively. It was also evaluated to show that the antioxidant activities of FA-g-NOCC and FA-g-CS were significantly higher than those of natural NOCC or CS. Animal experiments have shown that the CS derivatives visibly increased the activity of antioxidant enzymes in the serum and liver of aging mice induced by D-galactose and reduced the level of malondialdehyde. Their results suggested the potential of FA-g-CS in developing new antioxidants.

### 5.3. Applications

#### 5.3.1. Drug Delivery System

A drug delivery system (DDS) refers to the technical system that comprehensively regulates the distribution of drugs in an organism in space, time, and dose. Its goal is to achieve precise drug delivery and tumor-targeted release at the molecular level, thus increasing the utilization efficiency of drugs, improving efficacy, and reducing costs and side effects [108].

Polysaccharides have become one of the most promising polymers for developing various drug delivery systems due to their excellent properties. In order to tailor DDS, various forms have been developed in recent years, including nanoparticles, microparticles, tablets, gels, as well as films and membranes [80,109].

Studies have shown that grafting phenolic acid onto polysaccharides, such as CS, can also produce a new DDS [110]. According to Li et al. [58], a new type of DDS based on CS derivatives was synthesized by introducing FA to CS through free radicals with the grafting ratio of 102.93 mg FA/g. Bovine serum albumin (BSA) was encapsulated following a spray drying technique using CS-g-FA as wall material, affording microparticles of encapsulated BSA. An in vitro release study of microencapsulation showed that the excessive drug loading (c = 0.2 g) caused the drug density to be high, and the same area released more drugs, leading to a large number of water molecules entering the microspheres and forming more pores. This accelerated the early release of drugs, and the release effect was not obvious. However, when the drug loading was too small (c = 0.1 g), the low-density drug made it easy for water molecules to enter the microspheres and form more gaps. This would cause the initial release to be larger, and the time to reach the maximum release rate was 2.5 h. When the drug loading was 0.15g (c = 0.15g), the microspheres could be significantly release-sustained. At 15 h, the maximum BSA released from the microspheres was 72%. So, the slow release of BSA in Phosphate buffer (pH 7.4) over a period of 15 h showed the practicability of the embedding scheme. This study clearly demonstrated that CS-g-FA conjugate was a potential functionalized carrier material for drug delivery. However, this study had no evidence to suggest how this new drug carrier would help to improve the efficacy and reduce side effects.

#### 5.3.2. Emulsions for Nutraceutical Delivery Systems

*β*-Carotene (BC) possesses significant antioxidant properties. It is used in dietary supplements as well as in the treatment of degenerative diseases and several cancers [111]. BC contains unstable polydilute groups. To promote the chemical stability and bioaccessibility of BC, different food-grade delivery systems were designed by researchers [112,113,114]. For example, Yu and coworkers [115] evaluated the emulsifying performance and contribution of an FA-grafted curdlan conjugate (Cur-D-g-FA) to the chemical stability of BC. The results indicated that a 0.8% Cur-D-g-FA showed the best anti-droplet coalescence stability of a lotion from three aspects: droplet size, negative charge, and size distribution. Exposed to different environmental stresses, the emulsions stabilized by 0.8% Cur-D-g-FA could improve the chemical stability of BC and resulted in good bioaccessibility of BC in vitro. 

On the basis of this work, the team also studied the effect of FA-grafted carboxylic curdlan conjugates on BC storage stability [65]. FA-grafted carboxylic curdlan conjugates (Cur-8-g-FA and Cur-24-g-FA) were synthesized, and the grafting ratios of Cur-8-g-FA and Cur-24-g-FA were 223.03 mg FA/g and 115.63 mg FA/g, respectively. They prepared to obtain BC emulsions stabilized by FA–grafted carboxylic curdlan conjugates (Cur-8-g-FA/BC and Cur-24-g-FA/BC) and then determined the droplet sizes and zeta potentials of emulsions. The storage stability of BC-loaded pickering lotion was evaluated by periodically determining the content of BC. Results showed that their fairly good emulsion stabilities were demonstrated by comparing the droplet sizes and zeta potentials of BC emulsions stabilized with Cur-8-g-FA and Cur-24-g-FA. Furthermore, Cur-8-g-FA could avoid the decomposition of BC better than Cur-24-g-FA during storage in the emulsions. It showed that Cur-8-g-FA could serve as an alternative stabilizer to protect the structure of BC from dissociation. Thus, it could be used in the field of functional food.

## 6. Catechin–Polysaccharide Conjugate

### 6.1. Catechin

**Figure 3:**
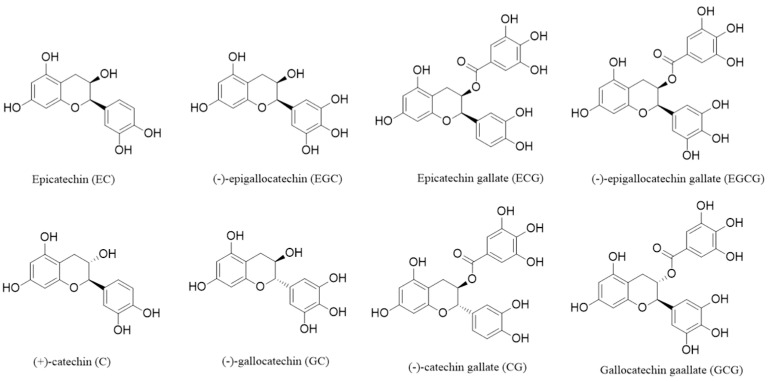
Chemical structure of eight isomers of catechin.

Catechin is a type of active natural phenolic substance extracted from plant species [116]. It has a structure of 2-phenylbenzopyran, composed of two benzene rings referred to as ring A and ring B, with a dihydrofuran structure in a ring C structure, and its third carbon connected with a hydroxyl group. Catechin mainly consists of four monomers and optical isomers. Their chemical structure is shown in Figure 3.

Catechin has a variety of physiological functions [117,118], such as antioxidation [119,120], anticancer [121], antimicrobial [122], hypoglycemic [123], lipid-lowering [124,125], and neuroprotection [126,127]. Researchers have performed structural modifications of these, mainly through physical and biochemical methods, to improve the physicochemical properties of catechins [128,129,130]. In recent years, the chemical combination of polysaccharides and catechins through graft copolymerization has been found to be effective for simultaneously enhancing the biological activity of polysaccharides and the stability of polyphenols [52,69,78,79,131,132,133,134].

### 6.2. Biological Activity

#### 6.2.1. Antioxidant Activity

The antioxidant properties of catechins grafted with different polysaccharides were evaluated, detecting multiple indicators over recent years. Liu et al. [52] reported that the free radical-mediated grafting of catechin onto *Tremella fuciformis* polysaccharide (TPS) was achieved by using a redox system, and the grafting ratio of the product was 265 mg CAE/g. The DPPH radical scavenging activity of TPS and catechin-g-TPS was enhanced in a concentration-dependent manner. Additionally, at the same concentrations, the scavenging activity of the polymer against DPPH radical was less than that of catechin. In terms of reducing capacity, which can also evaluate its antioxidant activity, the grafted copolymer had a stronger reduction ability than TPS.

The activity of conjugate was mainly dependent on the catechin fraction. Density functional theory (DFT) has been applied to study antioxidant compounds, and the calculations indicated that the antioxidant activity of substances was related to their molecular structure [135,136,137]. There are many electron-donating groups in the A and B rings of catechins, and they have a strong ability to capture free radicals. When catechins were grafted onto TPS, the antioxidation of TPS was significantly enhanced [137]. Identical conclusions were also reported by Cho [131].

Zhu et al. [79] studied the antioxidant function of catechin-grafted CS (catechin–g–CS) with a grafting ratio of 65.89 mg CAE/g. At the same concentration, the reducing power, hydroxyl, and DPPH radical scavenging ability of the graft copolymer were much higher than those of CS. The reduction ability is usually related to the presence of reductones with hydrogen-donating ability [57]. The intramolecular hydrogen bonds attenuated the hydrogen-donating ability of hydroxyl and amino groups. Therefore, the reduction ability of CS decreased. It was speculated that the strong reduction ability of catechin-g-CS might be due to the insertion of catechins that break the hydrogen bonds of CS, thereby increasing its hydrogen supply capacity.

#### 6.2.2. Antibacterial Activity

With the long-term application of antibiotics in the world, bacterial resistance has gradually become an issue of concern, so it is necessary to explore new antibiotics.

Research showed that the grafted polyphenol-polysaccharides had good antibacterial activity. Cho et al. [131] tested antimicrobial activity against methicillin-resistant *Staphylococcus aureus* (MRSA) and foodborne pathogens of catechin-g-CS conjugate (the grafting ratio was 22.17 mg CAE/g) using MIC value against two standard MRSAs, three standard MSSAs, and 15 clinical isolates. Cho et al. [131] tested antimicrobial activity against methicillin-resistant *Staphylococcus aureus* (MRSA) and foodborne pathogens of catechin-g-CS conjugate. They also tested the antimicrobial activity of the polymer and the unmodified CS against foodborne pathogens (three Gram-positive and six Gram-negative). The MICs of the catechin-g-CS conjugates were 64 µg/mL for *B*. *subtilis*, *E*. *faecalis*, and *L*. *monocytogenes*, which was less than the MIC value of unmodified CS. To summarize, catechin-g-CS conjugates showed better antibacterial activity than unmodified CS. The integrity cell membrane, outer membrane (OM), and inner membrane (IM) permeabilization experiments indicated that the graft could disrupt the cell membrane of germs, and it accelerated the release of *β*-galactosidase and increased the 1-N-phenylanphthylamine (NPN) uptake for both germs [138].

However, some research papers also reported that the antibacterial activity of the polyphenol-polysaccharide conjugates was slightly lower than that of CS, and the reason for the possibility was that the antibacterial activity also depended on strains, pH, and degree of substitution [78,139]. These studies verified that polyphenol-polysaccharide conjugates had good antibacterial activity.

#### 6.2.3. Antidiabetic Activity

Diabetes mellitus (DM) is usually a chronic disease with a combination of genetic and environmental factors. Among them, insulin-independent diabetes mellitus, also known as type 2 diabetes (T2DM), is currently an incurable fatal disease [140]. With the prevalence of diabetes worldwide, people urgently need a new generation of antidiabetic drugs. Natural polyphenols and polysaccharides, as well as their grafts, have some level of antiglycemic activity [141,142,143].

Zhu et al. [79] studied the antidiabetic potential activity of catechin-g-CS (the grafting ratio was 65.89 mg CAE/g). Using acarbose as control, which is a drug commonly used in the treatment of T2DM, the inhibitory effects of CS, catechin-g-CS, and catechin on glucosidase were studied. The inhibition rate of catechin-g-CS was the highest (72.45%), and the inhibition rates of CS, catechin-g-CS, catechol, and acarbose on α-amylase were 17.65%, 36.47%, 32.35%, and 62.94%, respectively. Among all test materials, acarbose showed the strongest amylase inhibition. The inhibitory effect of catechin on amylase was much lower than that of acarbose but higher than that of CS, which indicates some synergistic action exists between catechin and CS on α-glucosidase and α-amylase inhibitory effect. The results suggested that catechin-g-CS had a strong inhibitory effect against glucosidase and a mild inhibitory effect against α-amylase and possibly had contributed to the treatment of T2DM. However, there was a lack of an in-depth study on the anti-T2DM activity and potential mechanism of the graft copolymer.

#### 6.2.4. Neuroprotection Activity

Neurodegenerative diseases (NDs) damage the central nervous system (CNS). Due to structural damage or even apoptosis of neurons in the CNS, NDs can affect normal cognitive and motor functions in the human body [144]. Common NDs include Alzheimer’s disease (AD), Parkinson’s disease, amyotrophic lateral sclerosis, Huntington’s disease, and stroke [145,146]. Over the past decades, natural drugs have been isolated from natural medicinal plants, and many studies have been performed to investigate their role in NDs, such as AD, in terms of scavenging free radicals, inhibiting neuroinflammation and neuronal apoptosis, and enhancing the function of cholinergic neurons. Among them, polyphenols, alkaloids, and polysaccharides have been shown to have potential therapeutic effects on AD [147,148,149,150].

Researchers have focused on the neuroprotective function of polyphenol-grafted polysaccharides. Xu et al. [78] developed protocatechuic acid (PCA) grafted CS (PCA-g-CS) via free radical-mediated grafting reaction. Then, they studied neuroprotective effects against H_2_O_2_ and L-glutamic acid (GLU)-induced apoptosis in PC12 cells. The conclusion was that no great cytotoxicity of PCA-g-CS, natural CS, or chitooligosaccharides was found, even at high concentrations (0.8 mg/mL). The partially degraded native CS had little impact on the viability of the cells treated for 48 h. This indicated that the PCA components enhanced cell viability in the PCA-g-CS-treated cells rather than CS-derived glucosamine nutrients. Then, the neuroprotective effects of the copolymers against GLU-induced PC12 cell death were evaluated. The results showed that PCA-g-CS had comparatively good inhibition of GLU-induced excitotoxicity with the corresponding viability of 70.3 ± 3.1% and 75.9 ± 3.6%, respectively. Instead, different concentrations of CS had no significant impact on the survival of PC12 cells, indicating that grafted PCA played a very important role in the neuroprotective effects rather than CS. In short, the copolymer had certain protective functions in PC12 cells against H_2_O_2_- and GLU-induced oxidative damage, which might have application value in areas such as antioxidant drug release. However, deeper research, such as the mechanisms of neuroprotection and related signal transduction pathways, should be undertaken.

## 7. Other Polyphenol-Polysaccharide Conjugates

The above mainly summarizes the conjugates of GA, FA, and catechins with polysaccharides. In addition, some other polyphenols, such as proanthocyanidin, chlorogenic acid, and quercetin, were also conjugated to polysaccharides and had certain biological activities.

Proanthocyanidin is a large secondary plant metabolite called flavonoids, widely present in the flowers, fruits, and rhizomes of plants [151]. They have complex and diverse functions, and their antioxidant [152,153], antibacterial [154,155], anti-tumor [156,157], anti-inflammatory [158], and cardiovascular protection activities [159] are excellent. Proanthocyanidin–polysaccharide conjugates have been proven to have strong antioxidant activity and certain antibacterial activity against *E*. *coli* and *S*. *aureus* [160].

As another type of polyphenol plant extract, chlorogenic acid has many medicinal functions, such as anticancer [161], antioxidation [162], anti-inflammatory [163], and immune regulatory functions [164]. Research has shown that chlorogenic acid can be grafted onto CS by a free radical-mediated grafting method. Moreover, it was found that the grafting materials had an inhibitory effect on the growth of *E*. *coli*, *S*. *aureus*, and *B*. *subtilis*, indicating that they had the value of utilization as a postharvest fresh-keeping agent for food [93,165]. 

Additionally, quercetin was grafted onto starch, and the grafting products exhibited scavenging free radicals, inhibiting free radical formation and total antioxidant activity [166]. However, more efforts should be made to conduct in-depth mechanistic research to increase the possibility of practical applications of graft products in food, pharmaceutical, and biomedical fields.

**Table 1:** A summary of characterization methods, biological activities, and applications of polyphenol-polysaccharide conjugates mediated by free radicals.

Polyphenols	Polysaccharides	Characterization Methods	Biological Activities	Applications	References
FA	Curdlan	UV-vis, FT-IR, XRD, DSC, ^1^H NMR, ^13^C NMR,	DPPH scavenging activity; antioxidant capacity	Antioxidant additive	[47]
FA	Pectin	UV-vis, FT-IR, XRD, DSC, ^1^H NMR, ^13^C NMR, SEM, SEC–MALLS	DPPH scavenging activity; antioxidant capacity	Antioxidant additive	[48]
Catechin	*Tremella fuciformis* polysaccharide	FT-IR, ^1^H NMR, TGA, XRD, SEM	DPPH scavenging activity; reducing power	Antioxidant additive	[49]
GA, CA, FA	Carboxymethyl CS	UV-vis, FT-IR, ^1^H NMR, XRD	Superoxide, hydroxyl radical, H_2_O_2_, and DPPH scavenging activity; lipid peroxidation inhibition effect, reducing power	Antioxidant additive	[50]
CA, FA	CS	UV-vis, FT-IR, ^1^H NMR, ^13^C NMR, XRD, TGA	Superoxide, hydroxyl radical, and H_2_O_2_ scavenging activity; lipid peroxidation inhibition effect;	Antioxidant additive	[63]
GA	O–carboxymethyl CS	TLC, UV-vis, FT-IR, ^1^H NMR, XRD, SEM	DPPH radicals scavenging activity; reducing power; protective effect against H_2_O_2_-induced oxidative damage in RAW264.7 cells	Antioxidant additive	[56]
Quercetin	Starch	FT-IR, Fluorescence analyses, DSC	DPPH scavenging activity; total antioxidant activity; scavenging properties on peroxynitrite anion; α–amylase inhibitory activity	Drug delivery; drugs in the treatment of Alzheimer’s disease and diabetes	[166]
Catechin	CS	^1^H NMR, FE-SEM, TGA, XRD	Reducing power; hydroxyl and DPPH activity; in vitro anti-diabetic activity	Antioxidant additive; anti-diabetic agent	[76]
Banana condensed tannins	Inulin	UV-vis, FT-IR, XRD, TGA, ^1^H NMR, FE-SEM	Free radical scavenging activity; reducing power; the in vitro hypoglycemic activity	Antioxidant additive; anti-diabetic agent	[61]
GA, Catechin	CS		Total antioxidant activity; hydroxyl radical scavenging activity	Food preservatives	[51]
Catechin	CS	^1^H NMR	DPPH scavenging activity; protection ability against hydrogen peroxide-induced hepatic damage; inhibition activity against intracellular ROS formation and cell membrane lipid peroxidation	Antioxidant additive; antimicrobial	[128]
Catechin	Dextran	GPC, UV-vis, ^1^H–NMR, FT-IR	DPPH, ABTS, and hydroxyl scavenging activity; peroxyl radicals; the inhibition of lipid peroxidation; anticancer activity;	Drugs for pancreatic ductal adenocarcinoma	[129]
Catechin	Arabinoxylan	UV-vis, FT-IR, NMR, TGA, DTG	reducing starch digestibility and affecting gut fermentation	A novel dietary fiber	[67]
Vanilla acid, coumarin acid	CS	TLC, UV-vis, FT-IR	DPPH scavenging activity; total antioxidant activity; spectrum antibacterial activity against an array of bacteria;	Food preservative	[57]
Anthocyanin	CS	FT-IR, ^1^H NMR, XRD, TGA, DSC	DPPH, ABTS, hydroxyl radical scavenging activity; reducing power; antibacterial activity	Antioxidant additive; antimicrobial	[156]
Catechin	CS	UV-vis, FT-IR, ^1^H NMR	DPPH scavenging activity; excellent emulsifying activity and superior emulsifying stability	Natural food antioxidant and emulsifier	[131]
Catechin	Inulin	UV-vis, FT-IR, ^1^H NMR, SEM, DSC, TGA, XRD, Helix-coil transition assay	In vitro anti-diabetic activity	Anti-diabetic agent	[77]
FA	Carboxylic curdlan	UV-vis, FT-IR, SEC–MALLS, SEM, TGA	Antioxidant activity	Emulsions for β–carotene delivery	[62]
GA	CS	^1^H NMR, FT-IR, UV-vis, SEM	Osteogenic effects in murine bone marrow-derived mesenchymal stem cells (mBMMSCs)	Drugs for osteogenic effects	[78]
GA	CS	TGA	DPPH radical scavenging ability	Antioxidant active packaging film	[91]
GA	CS	UV-vis, FT-IR, ^1^H NMR, ^13^C NMR	Hypoglycemic activity	Anti-diabetic agent	[58]
FA	CS	FT-IR, UV-vis, DSC, XRD, SEM	A functional wall material for microencapsulation of BSA	Drug delivery	[104]
Chlorogenic acid	CS	UV-vis, FT-IR, ^1^H NMR	ABTS and DPPH scavenging activity; ferric and cupric reducing antioxidant power	Preservative agent and edible coating material in peach fruit	[90]
CA, Chlorogenic acid	CS	UV-vis, FT-IR, XRD, ^1^H NMR, ^13^C NMR	Antioxidant activity; antimicrobial activity	A post-harvest fresh-keeping agent for fruits and vegetables	[161]
GA	CS gallate	UV-vis, GPC, ^1^HNMR	Antioxidant activity; In vitro antimicrobial activity	Food packaging materials	[92]
Protocatechuic acid	CS	FT-IR, ^1^H NMR, XRD, UV-vis	Antioxidant activity; cytotoxic and neuroprotective assessment on PC12 cells	Antioxidative drug release; tissue engineering scaffolds materials	[75]
Epigallocatechin gallate	CS gallate	UV-vis, FT-IR, XPS, DSC, TGA	ABTS and DPPH scavenging activity; antibacterial activity	Antioxidant additive; antibacterial agent	[130]
GA	Chitin–glucan	^1^H NMR, FT-IR, XRD, SEM	ABTS and DPPH scavenging activity; antibacterial activity	Antioxidant additive; antibacterial agent	[93]
CA	CS	TLC, UV-vis, FT-IR			[54]
GA	CS	TLC, ^1^H NMR	DPPH and hydrogen peroxide scavenging capacity; Reducing power; good cytocompatibility against RAW264.7 mouse macrophages	Drugs in the treatment of diseases related to oxidative damage	[59]

## 8. Conclusions and Future Perspectives

In this review, a variety of polyphenol-polysaccharide conjugates prepared by the free radical-mediated method were reviewed. They are demonstrated to have the advantages of high efficiency, low cost, and greenness. For all types of conjugates, the most researched properties were enhanced antioxidant and antibacterial activities. Conjugates show potential in functional foods and drug development. Although an enormous amount of effort has been paid to the development of polyphenol-polysaccharide conjugates, more attention should be placed on the major prospective areas of study.

First, at present, the exact reaction mechanism of free radical grafting methods is controversial, and the evidence is still insufficient. There is also a lack of research on the synthesis mechanism and kinetics of polyphenol-polysaccharide conjugates, resulting in a low grafting rate. Further in-depth research is needed. Second, the detailed structural characteristics of the polyphenol-polysaccharide conjugates, such as the connection position and distribution mode of polyphenols, are still not yet known, which seriously hinders the establishment of the structure–activity relationship. In later stages, the conformation of the conjugates could be more intuitively explained through techniques such as 2D NMR. Third, most activity evaluations of the conjugates have been conducted in vitro, and more work should focus on in vivo biology. Last, the free radical grafting method is now widely applied to grafting modification of CS or functionalized CS, and it should be extended to other polysaccharides.

## Data Availability

Data is contained within the article.
